# Design of case report forms based on a public metadata registry: re-use of data elements to improve compatibility of data

**DOI:** 10.1186/s13063-016-1691-8

**Published:** 2016-11-29

**Authors:** Martin Dugas

**Affiliations:** 1 Institute of Medical Informatics, University of Münster, Albert-Schweitzer-Campus 1, A11, D-48149 Münster, Germany; 2 European Research Centre for information systems (ERCIS), Leonardo-Campus 3, 48149 Münster, Germany

**Keywords:** CRF, Data element re-use, MDR, CDISC ODM, ISO/IEC 11179, Information infrastructure

## Abstract

**Background:**

Clinical trials use many case report forms (CRFs) per patient. Because of the astronomical number of potential CRFs, data element re-use at the design stage is attractive to foster compatibility of data from different trials. The objective of this work is to assess the technical feasibility of a CRF editor with connection to a public metadata registry (MDR) to support data element re-use.

**Results:**

Based on the Medical Data Models portal, an ISO/IEC 11179-compliant MDR was implemented and connected to a web-based CRF editor. Three use cases were implemented: re-use at the form, item group and data element levels.

**Conclusions:**

CRF design with data element re-use from a public MDR is feasible. A prototypic system is available. The main limitation of the system is the amount of available MDR content.

## Background

Data management in clinical trials is resource-intensive because many case report forms (CRFs) need to be collected: on average, about 180 pages per patient [1]. This article refers to a CRF as an individual documentation form; therefore, each trial applies a set of CRFs. Despite these extensive documentation efforts, combined analysis of data from different trials is complicated. Variability of CRFs is a major challenge when merging data from different clinical trials. In principle, an astronomical number of different CRFs can be designed [2]. Therefore, the overlap of data elements between two CRFs is very small when these CRFs are designed independently, even if the medical subject matter is similar. This problem of related but not matching data structures has been described in the literature, such as regarding clinical decision support: ‘The largest barrier to linking knowledge-based medical decision support systems to heterogeneous [databases] is the variety of ways in which similar data are represented’ [3, page 204]. More standardised and compatible CRF data structures would enable integrated data analysis using different sources. In addition, data transfer from electronic health records to databases in clinical research would be facilitated [4]. One approach to foster more standardised CRFs is re-using data elements from a metadata registry (MDR) at the CRF design stage.

The objective of this work was to assess the technical feasibility of this approach (proof of concept) (i.e., development and implementation of a CRF editor with connection to an MDR and support for re-use of data elements). The system should be compliant with regulatory standards and apply a realistic set of data elements.

## Methods

### Metadata registry

ISO/IEC standard 11179 [5, page ﻿V] describes a metadata registry as ‘a database of metadata that supports the functionality of registration. Registration accomplishes three main goals: identification, provenance, and monitoring quality’. Identification is achieved by unique identifiers for metadata; provenance relates to sources of metadata. A data element according to this standard is specified regarding concept domain and value domain (i.e., a set of permissible values). Semantic information is needed for an MDR, because ‘an MDR manages the semantics of data’ [5, page V]. More specifically, an MDR enables researchers to compare objects (is a certain object already existing in the MDR?) and can ‘identify situations where similar or identical names are in use for administered items that are significantly different in one or more respects’ [5].

**Fig. 1:**
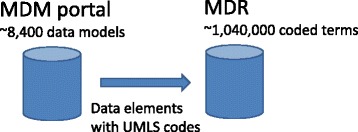
Data elements from the Medical Data Models (MDM) portal are automatically extracted from the MDM database and transferred to the metadata registry (MDR). *UMLS* Unified Medical Language System

The Medical Data Models (MDM) portal [6] is a public repository based mainly on CRFs. It is a registered European research infrastructure [7]. Semantic annotations (predominantly Unified Medical Language System [UMLS] codes [8]) are available for a subset of these data models and their data elements. Therefore, MDM was enhanced by an MDR software component which is processing only MDM data elements with UMLS annotations. Figure 1 presents the high-level architecture of the system. Basically, all data elements with UMLS codes are transferred from the MDM database to the MDR using Structured Query Language (SQL) database commands.

### Clinical Data Interchange Standards Consortium Operational Data Model

CRFs in clinical trials must comply with requirements of regulatory agencies. Standards of the Clinical Data Interchange Standards Consortium (CDISC) are being applied in this setting. Patient data items can be represented by CDISC Operational Data Model (ODM) [9], an open Extensible Markup Language (XML)-based transport format. Define XML (using CDISC ODM) is part of the U.S. Food and Drug Administration (FDA) Data Standards Catalog, which was announced to become mandatory for new drug applications by the end of 2016 [10]. Therefore, MDM and MDR are using internally ODM-compatible data structures.

### CRF editor

Electronic CRFs are designed with CRF editors. The CRF editor of the MDM portal was enhanced to support re-use of data elements. Re-use can be applied at different levels: re-use of complete documentation forms, re-use of item groups and re-use of individual data elements. This CRF editor is a web-based system; Asynchronous JavaScript and XML (AJAX) in combination with database commands (SQL) was applied to generate a list of suggested data elements for re-use during CRF design. Because of the large number of coded terms in the MDR (approximately 1,040,000), an asynchronous technique was applied to avoid performance issues. Re-use at the item group level and at the form level is provided by dedicated web services.

## Results

### Search function for MDR

**Fig. 2:**
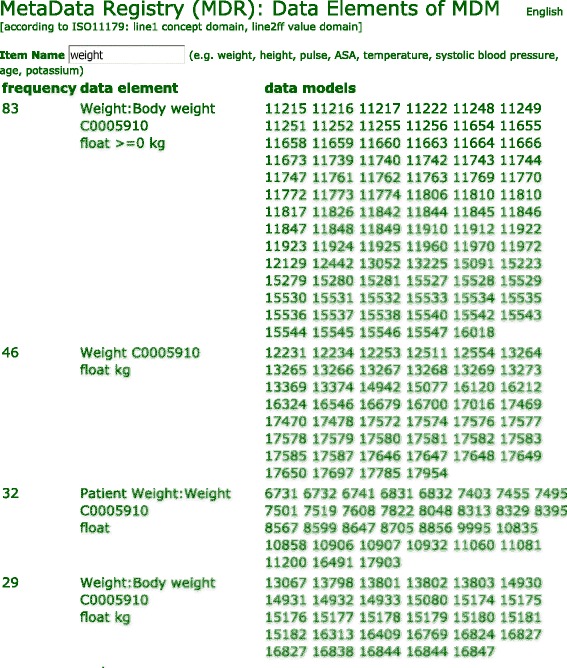
Search function of the metadata registry. Available data elements named *weight* are presented. The most frequent element is ‘weight:body weight’ with Unified Medical Language System code C0005910 (concept domain), measured as floating point number in kilograms (value domain). By clicking on a data model identifier (*right column*), all details about data element context are provided. *MDM* Medical Data Models

A prototypic MDR implementation is available at http://mdr.uni-muenster.de. Figure 2 presents the graphical user interface (GUI). When an item name is entered, a table of matching data elements from the MDR is displayed. It is ordered by frequency and contains links to respective data models. By this means, users can review the context of each element. For each data element, a short name and more detailed text are provided, separated by a colon. The language of these texts can be selected. At present, most data elements are available in English and German. The concept domain is characterised by a UMLS code. The value domain is described by data type and, if appropriate, by unit, minimum/maximum or a list of permissible values.

Overall, approximately 240,000 data elements with approximately 1,040,000 coded terms (UMLS codes) are available in the MDR. The number of terms is higher than the number of elements because each element can be translated into several languages (e.g., English, German, Dutch). This GUI can be used to look up data elements in the MDR.

### Re-use of data elements at form, item group and item levels

**Fig. 3:**
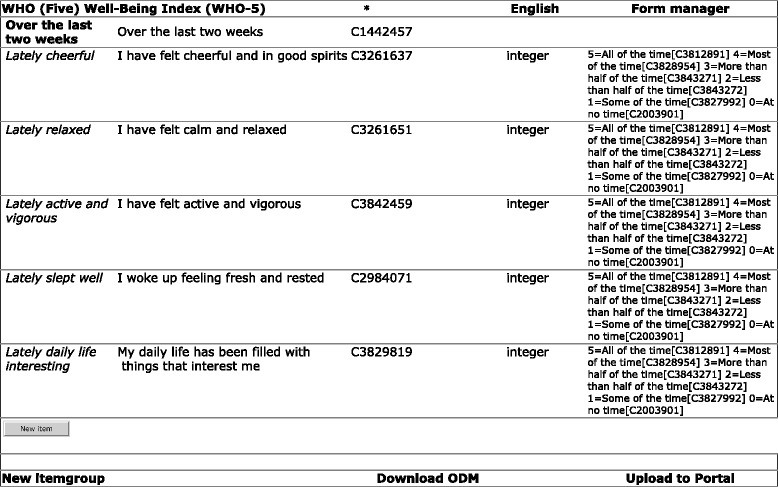
Re-use at the form level. The complete form (WHO-5 questionnaire [22] in this example) can be re-used via ‘Download ODM’ (Operational Data Model) and imported into a new case report form system

A prototypic implementation of a CRF editor with re-use functionality is available at http://odmeditor.uni-muenster.de. Re-use of data elements during CRF design can occur at different levels. A study consists of a set of CRFs. In principle, a whole CRF from a previous study could be re-used for a new study. An example of this use case is provided in Fig. 3.

**Fig. 4:**
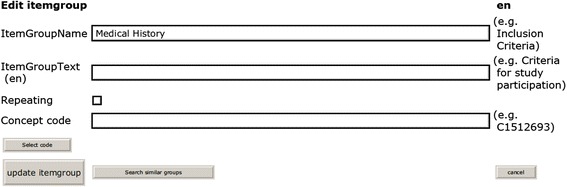
Re-use at the item group level. Using the ‘Search similar groups’ button (*bottom left, second button*), similar item groups for ‘Medical History’ can be identified. (For this search result, see Fig. 5.)

**Fig. 5:**
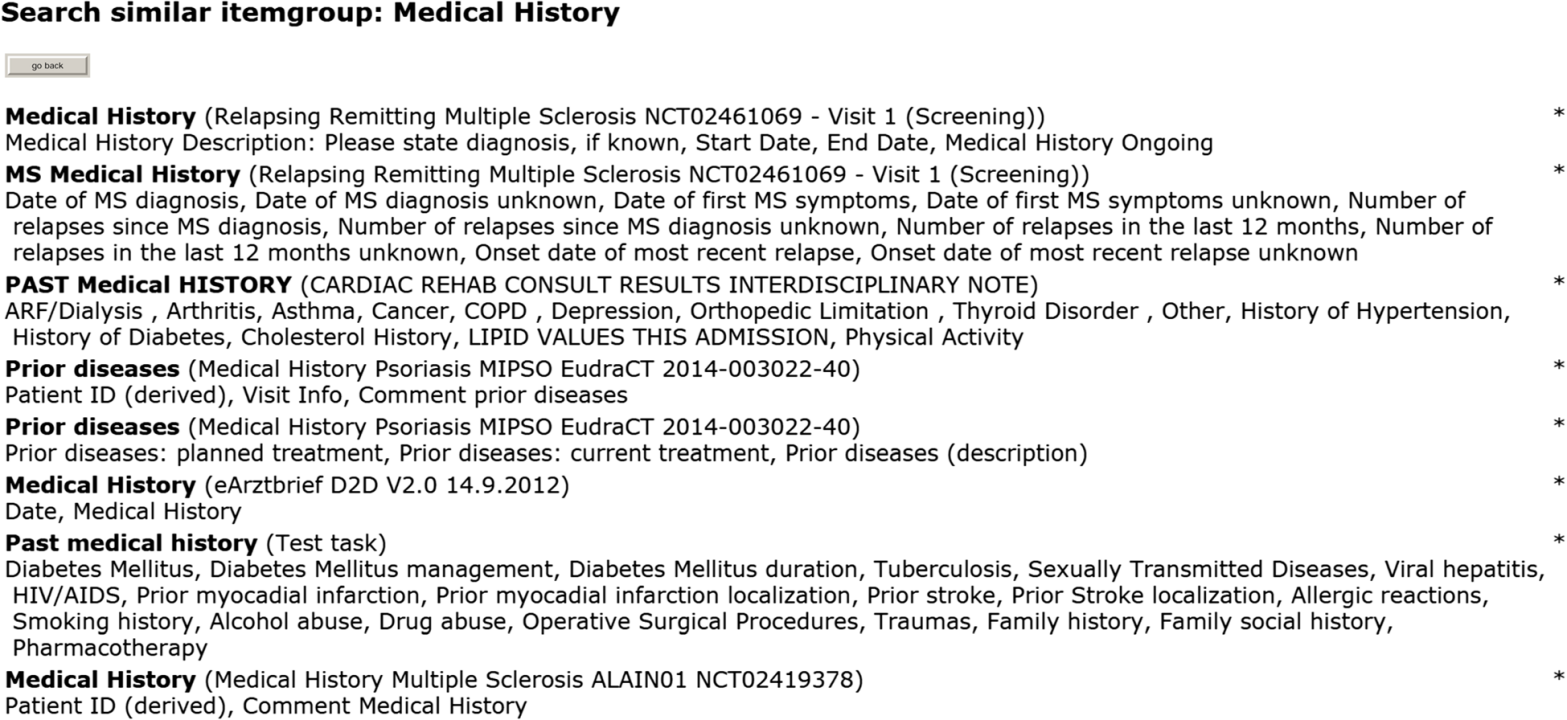
Search result for similar item groups (initial section). By clicking on an appropriate item group, all its elements are copied into the new case report form

Another use case is re-use of an item group from a previous study (i.e., a list of related data elements). Figures 4 and 5 present screenshots from the prototypic implementation. Specific search terms for item groups should be applied because generic search terms such as *Physical examination* can produce a long list of results.

**Fig. 6:**
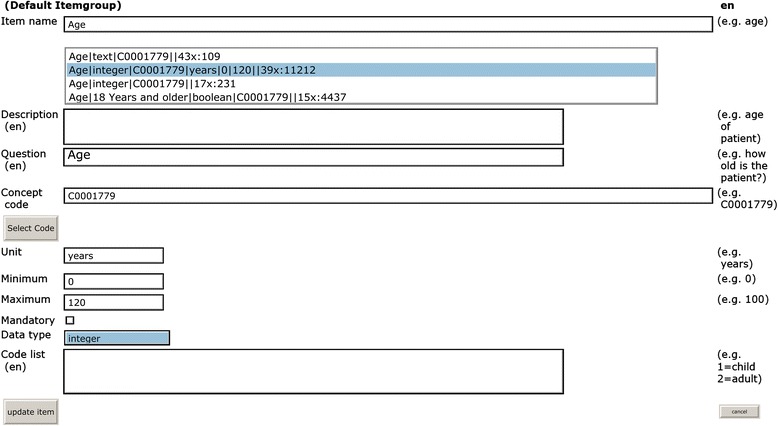
Re-use at the data element level. When an element name (e.g., ‘Age’) is entered, similar elements from the metadata registry are presented and can be copied into the new case report form

The third use case is re-use of data elements at the element level, illustrated in Fig. 6. A catalogue-based search of data elements is not efficient, because there are more than 240,000 elements in the MDR; the usability of the system would be limited because finding and selecting an appropriate data element would require many clicks and keystrokes. Therefore, an automated approach was implemented. While the user enters a new data element, a list of matching elements for re-use is generated and updated. A data element for re-use can be selected at any time, or these suggestions are ignored and a new element is defined from scratch.

In principle, it is possible to predict the next element of a new CRF on the basis of context. The next element after surname is frequently first name; aspartate transaminase (AST) is documented often together with alanine transaminase (ALT). (AST and ALT are both liver parameters.) This contextual information (what data elements are used frequently on the same CRF like a given element?) can be extracted from the MDM portal. In the current prototype, information from two preceding data elements is analysed to generate suggestions for the next element.

## Discussion

The theoretical benefits of re-using data elements for medical documentation have been described before [4, 11]. CRF quality could be improved, such as with fewer typing errors by re-using high-quality CRFs. CRF design could be more efficient, such as through less manual input by re-using code lists. From my perspective, the aspect of standardisation by re-use is of interest. It is known from the literature that an astronomical number of CRFs can be designed. This leads to incompatible data in different studies (i.e., not suitable for data integration). Therefore, re-use of data elements for CRFs seems attractive to avoid incompatible modelling of similar items; for example, a pain scale with four levels generates data incompatible with that from a pain scale with five levels. This should be avoided wherever possible at CRF design stage. In the long run, the proposed re-use of data elements would also be beneficial for meta-analysis because more homogeneous data collection would be fostered and compatibility of patient data would be improved. Previous work [12] has shown that the 100 most frequent medical concepts cover 25% of all concept occurrences in clinical trials. However, owing to the semantic complexity of medicine, there is a large number of rarely used medical concepts in clinical trials.

A prerequisite for data element re-use is access to elements from previous studies. Open metadata is demanded by scientists [13, 14] but is not (yet?) the norm; therefore, currently, the vast majority of CRFs are not available to the scientific community. In recent years, more and more data elements are being made available via various MDRs, such as the cancer Data Standards Registry and Repository of the National Cancer Institute [15], the National Institute of Neurological Disorders and Stroke project [16], the Clinical Element Model [17] or the Metadata Online Registry of the Australian Institute of Health and Welfare [18]. A special feature of the MDM [6] is provision of complete CRFs (i.e., data elements with relationship to other elements).

In this context, the objective of this work was to develop, for the first time to my knowledge, as a proof of concept a CRF editor with connection to an MDR and support for re-use of data elements. This prototype is now available to the scientific community. It applies relevant international standards, in particular ISO/IEC 11179 for MDRs and CDISC ODM, which is supported by regulatory agencies.

### Limitations and future work

This prototypic CRF editor has limitations. Most important, available data elements for re-use are derived from only about 8400 forms from the MDM portal. There are more than 227,000 registered trials [19] with approximately 180 pages each (i.e., about 41 million CRFs), corresponding to approximately 1.6 billion data elements (assuming, on average, 40 data elements per CRF). If current initiatives for more transparency in clinical trials [20, 21] are successful, public information infrastructures of data elements for CRFs will grow further. When more complete MDRs for CRFs are available, the approach of CRF design with data element re-use can be evaluated in realistic clinical research settings. Then it should be determined what proportion of CRF data elements can actually be re-used. This will also contribute to assessment of the benefit of data element re-use for data integration.

## Conclusions

CRF design with data element re-use from a public MDR is feasible. A prototypic system is available. The main limitation of the system is the amount of available MDR content.

## Abbreviations

AJAX: Asynchronous JavaScript and XML
ALT: Alanine transaminase
ASA: System for assessing the fitness of patients before surgery by AmericanSociety of Anesthesiologists
AST: Aspartate transaminase
CDISC: Clinical Data Interchange Standards Consortium
CRF: Case report form
FDA: U.S. Food and Drug Administration
GUI: Graphical user interface
MDM: Medical Data Models
MDR: Metadata registry
ODM: Operational Data Model
SQL: Structured Query Language
UMLS: Unified Medical Language System
XML: Extensible Markup Language
